# Reconfigurable origami-inspired multistable metamorphous structures

**DOI:** 10.1126/sciadv.adk8662

**Published:** 2024-05-29

**Authors:** Chunlong Wang, Hongwei Guo, Rongqiang Liu, Zongquan Deng, Yan Chen, Zhong You

**Affiliations:** ^1^ School of Mechanical Engineering, Tianjin University, Tianjin 300350, China.; ^2^ State Key Laboratory of Robotics and System, Harbin Institute of Technology, Harbin 150001, China.; ^3^ Department of Engineering Science, University of Oxford, Oxford OX1 3PJ, UK.

## Abstract

Origami-inspired metamorphous structures can adjust their shapes and mechanical behaviors according to operational requirements. However, they are typically composed of nonrigid origami, where required facet deformation complicates actuation and makes them highly material dependent. In this study, we present a type of origami metamorphous structure composed of modular bistable units, each of which is a rigid origami. The elasticity within the origami creases and switching of mountain and valley crease lines enable it to have bistability. The resultant metamorphous structure has multistability, allowing it to switch among multifarious configurations with programmable profiles. This concept was validated by potential energy analysis and experiments. Using this concept, we developed a robotic limb capable of both lifting and gripping through configuration changes. Furthermore, we used the origami units to construct a metamaterial whose properties could change with the variation of configurations. These examples demonstrate the concept’s remarkable versatility and potential for many applications.

## Teaser

An origami metamorphous structure with multistable layouts is proposed for multipurpose applications from robotics to materials.

## INTRODUCTION

Metamorphous structures have always attracted interests from scientists and engineers. One essential feature of these structures is their shape changing ability and accompanied change of mechanical properties. More recent ones are programmable according to requirements (*1*–*7*), and some are even adaptive to their operational environment (*8*–*13*). There exists a family of metamorphous structures that are inspired by origami objects because these simple objects often offer rich geometrical features unmatched by other structures. Origami morphing structures have been extensively developed in fields such as robotics (*14*–*21*), mechanical metamaterials (*12*, *22*–*26*) and aerospace deployable structures (*27*–*29*). To achieve morphing, some origami structures rely on the switch of mountain and valley assignments of creases to alter folding motions (*30*–*34*), whereas others use flexibility of the materials to obtain multistable metamorphous structures (*5*, *13*, *19*, *24*, *25*, *35*–*37*). At present, most such structures adopt a particular shape-changing mechanism or design from which almost all functions are derived. In other words, once the design of a structure is determined, rarely can it acquire new configurations (and therefore new functionalities) beyond those associated with the original design (*10*, *11*, *24*, *25*, *38*–*42*). There is a distinct lack of reconfigurability.

Here, we present a new family of programmable and reconfigurable modular multistable metamorphous structures. The constitutive unit of the structure is composed of a pair of origami cells that is bonded together. Kinematically the unit is a rigid origami with a motion bifurcation. However, we are able to show that the unit becomes bistable when the active creases are elastic. Assemblies of such units lead to metamorphous structures that can change their shapes and are reconfigurable through switch of stable configurations of their constitutive units. As a result, the mechanical properties and even its functions can be altered within the same structure. The units are scalable and the concept is modular, and multiple stable configurations of the structures are only related to the stiffness of the creases and the rest states of origami cells at which they are manufactured and thus, independent of the materials of the rigid facets. These features provide the possibility of creating structures of all scales for target applications. To demonstrate this, we built, first of all, a robotic limb capable of reconfiguring its operational profiles automatically to lift or hold weights of various shapes, and second, a programmable mechanical metamaterial capable of altering its mechanical properties such as Poisson’s ratios. Prototypes were fabricated to validate these concepts. We expect that our multistable metamorphous structures would facilitate the development of advanced metamaterials and reconfigurable morphing structures.

## RESULTS

### Geometry of the cells and unit

**Fig. 1.**
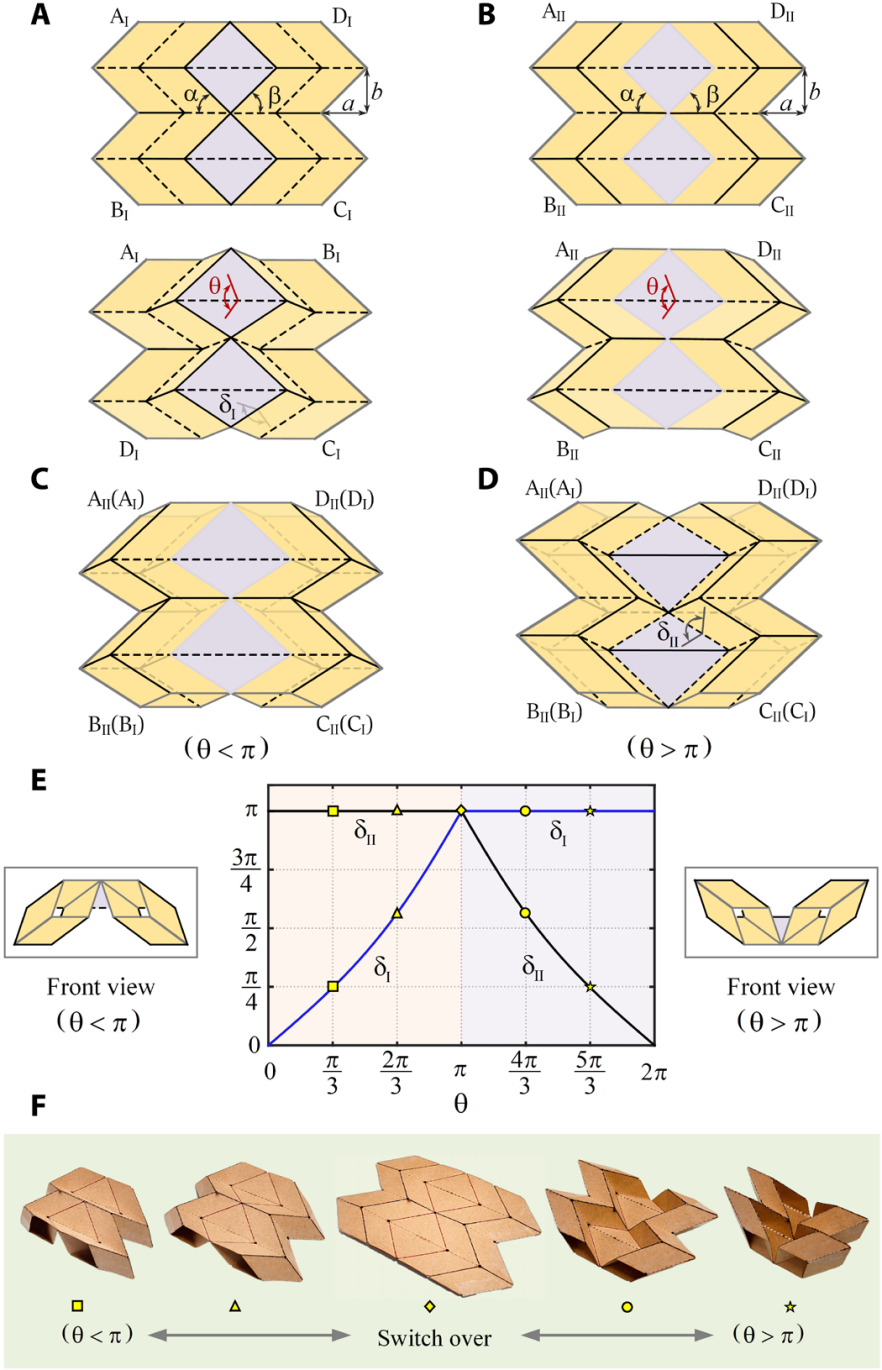
Bistable origami unit. (**A**) Crease pattern and partially folded shape of cell I where *a* = *b* = 15 mm. (**B**) Crease pattern and partially folded shape of cell II of the same dimension. (**C**) Origami unit created by stacking cell II on top of cell I and bonding the shaded central rhombuses and left and right edges together, respectively. (**D**) Configuration of the origami unit when cells I and II are switched over. (**E**) δ_I_ and δ_II_ versus θ plot. θ = π is the motion bifurcation state where both cells are completely flat. (**F**) Transition process between different configurations of the unit made from card (movie S1).

The origami unit is a combination of two similar origami cells. The crease pattern of the first one, referred to as cell I, is given in Fig. 1A. Black solid lines are mountain creases, whereas dashed lines are valley creases. All the inclined creases are parallel, and the rest are horizontal. It is a rigid origami pattern with a single degree of freedom (DOF). θ, the dihedral angle between two triangular facets on either side of the diagonal of the central rhombuses, could be treated as an input that uniquely determines the folded shapes of the cells, as given below the pattern. The key geometrical parameters are dimensions *a* and *b*. Here, we first take *a* = *b*, and α = π/4. Moreover, we let β = α. The crease pattern for cell II is shown in Fig. 1B. It has the identical geometry parameters but opposite fold assignments to those in cell I except those around two shaded central rhombuses. The edges of the rhombuses are dormant creases. To meet the rigid foldable conditions, the creases along their diagonals become valley creases. Its partially folded shape is displayed beneath the pattern. The behavior of both origami cells is demonstrated in movie S1.

The origami unit, shown in Fig. 1C, is created by stacking cell II on top of cell I and then bonding the shaded central rhombuses of two cells and their corresponding left and right edges together, respectively. This is possible when θ angles in both cells are made identical. The unit remains to be a flat-foldable rigid origami with a single DOF with θ angles, now merged together, being taken as the input, and it deploys and collapses easily.

The relationships between θ and δ_I_ as well as δ_II_, which are the dihedral angles between a triangular facet and its adjacent parallelogram facet along one of the edges of central rhombuses for cells I and II, respectively, can be obtained (text S1). We plot them in Fig. 1E. It can be seen that when θ = π, both cells are completely flat. Decreasing θ results in cell I being folded up with δ_I_ reducing steadily along the blue path while δ_II_ remaining at π. If, instead, θ is increased, i.e., the valley creases along the diagonal of rhombuses in cell I are inverted to mountain creases, the dormant creases along the edges of the central rhombuses are activated. δ_II_ decreases along the black path, while δ_I_ stays unchanged as a constant π, indicating that the creases along the edges of rhombuses of cell I become dormant. Effectively, cells I and II are switched over, as shown in Fig. 1 (C and D). This transition only occurs at θ = π. In other words, θ = π is a kinematic bifurcation point.

A card model of the unit was built in which the diagonal creases of the two central rhombuses were cut open to reduce the influence of the material thickness as the cells are bonded together at these rhombuses when forming the unit, making these areas rather thick. The transition of the unit between different states is shown in Fig. 1F and movie S1. Two observations were made. First, by poking the convex central vertex of the unit in a configuration close to the bifurcation state with a probe, the cells snapped from one configuration to another. Second, although it is theoretically possible both cells deploy to an identical shape after passing the bifurcation state, as indicated in Fig. 1D, this never occurred in practice for reasons which we shall discuss next.

### Bistability of the unit

In most origami models, the creases are not perfect mechanical joints that can rotate freely. Rather, they exhibit certain stiffness. If the creases are assumed to behave like linear elastic torsional springs with a stiffness *k* per unit length whereas the facets are rigid (*5*, *32*, *38*), the potential energies for each cell are $\Pi_{C-I}=\sum_{i=1}^{m}\frac{1}{2}{\mathrm{kL}}_{i}\;{\left(\phi_{i}-\phi_{i0}\right)}^{2}$ and $\Pi_{C-\text{II}}=\sum_{j=1}^{n}\frac{1}{2}{\mathrm{kL}}_{j}\;{\left(\phi_{j}-\phi_{j0}\right)}^{2}$ , where Π_C-I_ and Π_C-II_ represent the potential energy of cell I and cell II, respectively, *m* and *n* are total number of creases in each cell, *L* is the length of a crease, and ϕ and ϕ_0_ are final and initial rest dihedral angles of a crease, which include θ and δ used earlier to describe motions of cells. Note that the diagonal creases of the two central rhombuses are not included in the calculation since they were slit open when forming the unit. Hence, the total potential energy of a unit Π = Π_C-I_ + Π_C-II_ + Π_E_ in which Π_E_ is the potential energy of the edge creases formed when the edges of two cells are bonded together, and this energy can be calculated using a formula similar to Π_C-I_ or Π_C-II_. Obviously, the rest dihedral angles ϕ_0_ are set by the initial state of a cell (text S2).

**Fig. 2.**
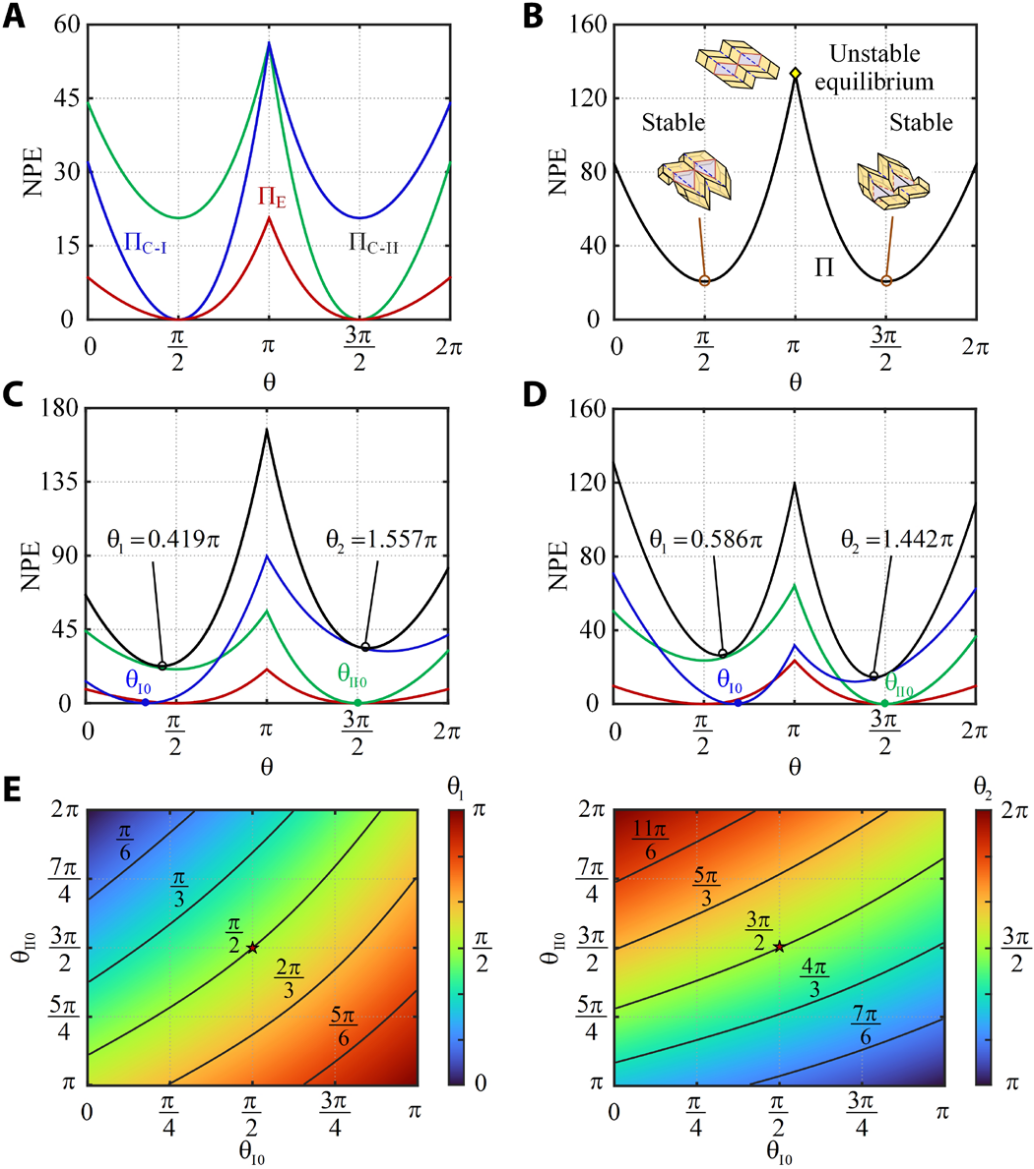
Potential energy of the origami unit. (**A**) NPE versus θ curves for cell I with θ_I0_ = π/2 (blue), cell II with θ_II0_ = 3π/2 (green), and edge creases (red). (**B**) NPE of the origami unit. (**C**) NPE versus θ curves for each cell and the unit composed of these cells where θ_I0_ = π/3 and θ_II0_ = 3π/2. (**D**) NPE versus θ curves for each cell and the unit composed of these cells where θ_I0_ = 2π/3 and θ_II0_ = 3π/2. (**E**) Contours of θ_1_ and θ_2_ of origami units with respect to different initial rest angles θ_I0_ and θ_II0_.

Now consider cell I with a rest angle θ_I0_ = π/2. Its normalized potential energy (NPE) versus θ curve is plotted in blue in Fig. 2A. It is noticeable that the energy curve has two wells at θ = θ_I0_ = π/2 and θ = 3π/2. This indicates that cell I is a bistable structure with two stable states. If cell II is constructed exactly the same as cell I, i.e., θ_II0_ = 3π/2 because of the way that we define θ in cell II, it will also exhibit two stable states, one at θ = θ_II0_ = 3π/2 and the other at θ = π/2, drawn as the green curve in Fig. 2A. The second stable state of cell II matches the first stable position of cell I. Hence, we can fold cell II to reach the configuration with θ = π/2 and then bond both cells together to create a unit. The rest angles at the edge creases of the unit are set as the angles when bonding of the edges takes place, and the associated energy is plotted in red in Fig. 2A. The overall potential energy of the unit, Π, is given in Fig. 2B. It is symmetrical about the bifurcation state (θ = π), and Π still has two wells at θ_1_ = π/2 and θ_2_ = 3π/2, respectively, indicating that there are two stable states for the unit as expected. If we select θ_I0_ = π/3 and θ_II0_ = 3π/2, the overall energy curve is no longer symmetrical, but it remains to have two wells at θ_1_ = 0.419π and θ_2_ = 1.557π, different from those angles of each cell (see Fig. 2C). When θ_I0_ = 2π/3 and θ_II0_ = 3π/2, the energy curve shown in Fig. 2D is similar to those of other units where two energy wells appear at θ_1_ = 0.586π and θ_2_ = 1.442π. It can be concluded that if the creases behave like a linear elastic rotational hinge, the origami unit becomes a bistable structure whose stable states are related to but slightly different from the stable states of each cell. Figure 2E gives contours of the angle θ at two stable states when different initial rest states of the cells are selected.

For all units, there always exists a localized NPE peak when θ = π, e.g., the one shown in Fig. 2B. However, it is unlikely that the switch between two stable states will follow the NPE curve over this peak. Any small perturbation will result in the structure rapidly snap from one stable state to another, similar to the snap-through behavior in buckling of curved elastic beam systems (*43*). This is confirmed by our observation of the card model shown in Fig. 1F and movie S1. Moreover, the two cells will never take the same shape as doing so will require higher NPE (text S2).

### Experimental validation of bistable units

**Fig. 3.**
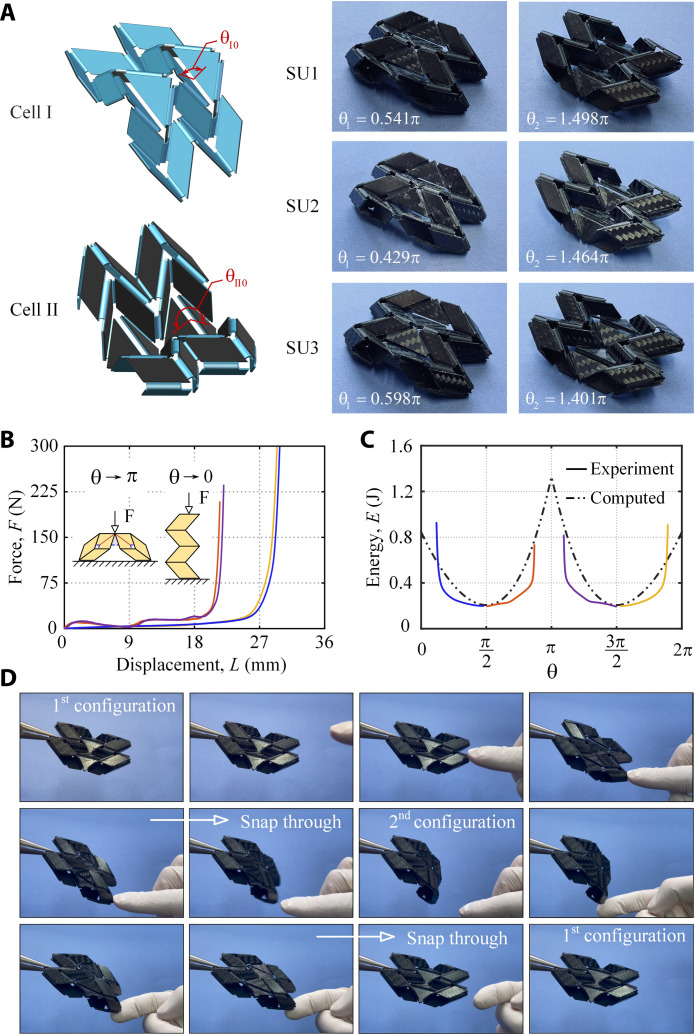
Fabrication and experimental validation of the bistable units. (**A**) Manufactured unstrained forms of cells I and II and resulting units. (**B**) Measured forces versus displacement in the force direction for SU1. (**C**) Energy plots for SU1. (**D**) Transition process between two stable states of the first prototype when pushed by a probe. Transition took place in the first and second photos of the second row (movie S2).

Three sample units, SU1, SU2 and SU3, were constructed. First, three sets of cells were manufactured in their unstrained forms using a 0.6-mm-thick elastomeric material, in which θ_I0_ = π/2, π/3, and 2π/3, respectively, while θ_II0_ was kept unchanged at 3π/2, and 0.4-mm-thick carbon fiber laminate sheet was cut and bonded to each facet to maintain rigid origami and confine all deformation to the creases (see Fig. 3A). Each cell II was then deformed to a shape with its θ close to θ_I0_ using a rig and then bonded with cell I to form a unit. The edges of both cells were made slightly wider so that the corresponding edges could be bonded together to form edge creases of the unit (text S3).

We then measured the angles corresponding to two stable configurations of the units, whose values are given in Fig. 3A. The actual angles are close to the theoretical ones with a maximum error of 16.7% (θ_2_ of SU2).

A total of four experiments were conducted for each unit. Starting from each of its two stable states, a unit is compressed to move away from this state either by either a lateral force (increasing θ), or a set of in-plane point forces (decreasing θ) as indicated in Fig. 3B. The energy curves were then obtained through integration of the measured forces over the corresponding displacements. The NPE versus θ plot of SU1 is given in Fig. 3C. The trend of experimental curves matches well with that of analysis, although the computed NPE is overestimated. This is because creases of the prototypes had finite width, whereas the theoretical width of the creases is zero, and thus, the actual model is less stiff than the analytical model.

Figure 3D shows the transition process of the first prototype by an external stimulus (movie S2). It was noticed that the unit never folded flat because the flat configuration does not correspond to any stable state, and a snap-through process took place in less than 1 s as shown in the first and second photos of the second row. Two stable states of the unit were symmetric to each other.

### Multistable robotic limb made from the origami unit

**Fig. 4.**
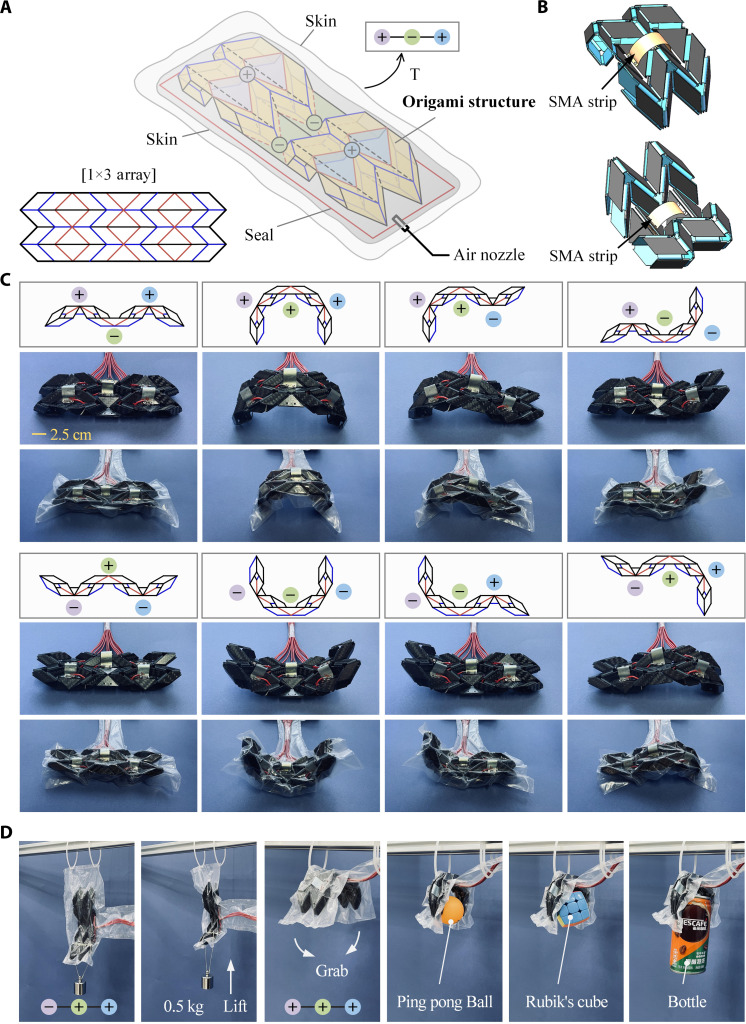
Multistable robotic limb. (**A**) Origami crease pattern and schematic diagram of the partially folded robotic limb. (**B**) Arrangements of SMA strips in an origami unit. (**C**) Front views and the limb in eight stable configurations and its corresponding relaxed and contraction (folded) states. (**D**) The robotic limb lifts a weight (movie S4) or grabs objects (movie S5).

The origami unit is scalable and could be used as building blocks for robots. Here, we use a robotic limb to demonstrate its vast possibilities. Figure 4A shows the origami pattern and its partially folded state for a limb consisting of a chain of three units where two units are bridged together by an inverted unit. Note that, although we state that there are three units, some facets of the neighboring units are actually overlapping. In its front view, we use ⊕ or ⊖ to mark the profile when a unit is convex up or down. This multistable robotic limb consists of three main components: the origami skeleton, a set of arc-shaped strips made from shape memory alloy (SMA) that are mounted on the central vertex of each unit for reconfiguration, and a flexible air-tight skin over the entire structure and a pump used to deploy the skeleton in each configuration (text S4). Figure 4B shows the arrangements of SMA strips in a unit in which a pair of them is installed on either side of the central vertex, and thus, there were a total of six SMA strips. Heating up one could force the vertex to invert, resulting in the unit to switch from ⊕ to ⊖ configurations, or vice versa (movie S3). Unlike conventional actuators, an SMA strip does not provide continuous control. Rather, it only provides an impulsive moment to let the unit snap from ⊕ to ⊖ and thus, much simpler in design. Once the skeleton enters into a particular configuration, e.g., ⊖-⊕-⊖, the force generated by the SMA actuators could be removed without causing the configuration change because each configuration is mechanically stable. However, its shape can further change by altering the pressure in the bag. Since the limb has three units, it has a total of eight configurations (2^3^). Figure 4C shows those configurations.

The robotic limb has many potential applications. For instance, when the limb is in ⊖-⊕-⊕ configuration, it can lift a weight (movie S4). To act as a gripper, it needs first to reconfigure from ⊖-⊕-⊖ to ⊕-⊕-⊕ configuration through SMA strips and then it grabs an object through its shape change activated by withdrawing air from the airbag. The entire process is demonstrated in both Fig. 4D and movie S5. In comparison with existing artificial limbs with a single operation mode, this multistable pneumatic-driven limb provides more operational programmability. It achieves, for instance, lifting and gripping modes in one assembly. Moreover, the gripper can also easily cope with objects of various shapes and weights.

### Reprogrammable mechanical metamaterials

Not only could the bistable units be placed in series to form an artificial limb, it could also be extended laterally to create reconfigurable and programmable mechanical metamaterials to achieve desirable features such as negative Poisson’s ratio, variable stiffness, and shape transformations.

**Fig. 5.**
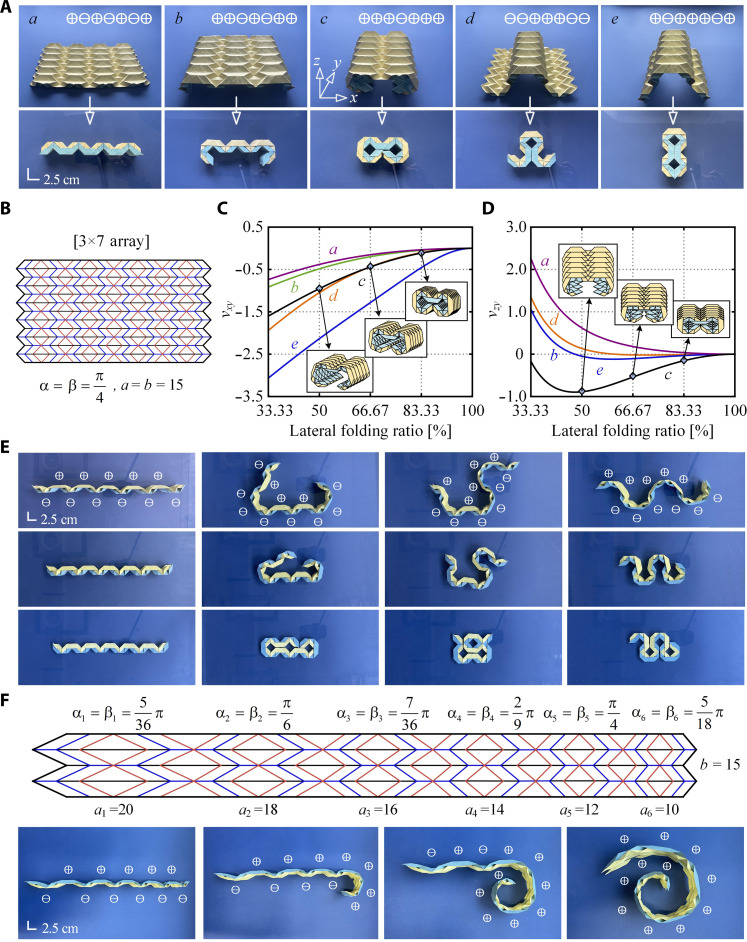
Reconfigurable and programmable origami metamaterials. (**A**) Five selected symmetrical stable configurations of this metamaterial and corresponding fully compressed (flat folded) states. (**B**) The origami pattern of the metamaterial. (**C**) Poisson’s ratios ν*_xy_* versus lateral folding ratio plots. (**D**) Poisson’s ratios ν*_zy_* versus lateral folding ratio plots. (**E**) Four possible configurations for the same origami structure. Each column shows the deployable sequence of the structure in a particular configuration. (**F**) The reconfigurable origami structure in a more general form where each unit is different.

Figure 5A shows an origami metamaterial built using 0.12-mm-thick cards, whose pattern consists of three columns of units laterally. In the longitudinal direction, these columns of units are arranged similarly to that of the multistable limb. The total number of columns is seven, and thus, there are 21 units. The origami pattern is given in Fig. 5B. In its original state, the metamaterial takes the shape of ⊕, ⊖, ⊕, ⊖, ⊕, ⊖, and ⊕. Note that unit within the same column behave exactly the same, i.e., they are either ⊕ or ⊖, otherwise, the mountain and valley rules would be violated. It has therefore a total of 128 stable states (2^7^) (movie S6) among which five symmetrical stable configurations of the same metamaterial are displayed in Fig. 5A. The interchangeable configurations could be reached once external stimuli are applied when the origami is close to its flat shape. We have investigated the Poisson’s ratios of the metamaterial, which are defined as functions of the folding ratio in the *y* direction (text S5). We placed an acrylic plate on top of the metamaterial and two weights were placed on top of the plate to impose a compressive force in the *y* direction. The Poisson’s ratio, ν*_xy_* and ν*_zy_* were then obtained, which are plotted in Fig. 5 (C and D). ν*_xy_* was always negative and monotonically decreasing with the increase of the folding ratio, while ν*_zy_* could be either positive or negative according to the folding ratio.

The number of distinct configurations of a reprogrammable mechanical metamaterial depends on the number of units in series. It is C(*n*, 2) where *n* is the number of the units. Figure 5E shows a single structure made from 11 units in four configurations out of 55 possible ones. Each column shows the deployable sequence of the structure in a particular configuration. Instead of using solely symmetrical units where α = β (see Fig. 1A), unsymmetrical ones could also be included where α ≠ β. An example is shown in Fig. 5F with its four configurations amongst many others. Use of unsymmetrical units can lead to spiral profile, avoiding collision between first and final units in a chain of units.

## DISCUSSION

The reported work provides an origami-based building block for multistable metamorphous structures that process many stable configurations. This feature allows the structures to be reconfigurable from one set of motions to a completely different set. Once the reconfiguration takes place, the mechanical behavior also changes accordingly with the new stable structural layout. We have demonstrated a modular structural concept where the geometric design of a unit can be efficiently exploited to customize its two stable configurations, and the behavior of individual units gives rise to the overall reconfigurable feature that is rare in such structures. Our findings are validated by experiments. It was observed that the kinematic behavior of our physical models was broadly close to the rigid origami behavior due to the fact that they had a rigid folding–based designs, although various materials of different stiffness were used. The method presented in this work could be extended both to larger and smaller scales metamorphous structures. As our mechanical metamaterials are multistable, they can also be designed to achieve target geometry configurations and physical properties. Although we only used two examples to show the potential of our modular metamorphous structures, our research offers a new design paradigm for the reconfigurable shape morphing structures and metamaterial architecture that can potentially be used to realize multifunctional robotic systems, bioinspired morphing mechanisms, and advanced metamaterials.

## MATERIALS AND METHODS

The bistable origami unit (Fig. 1) was folded using card paper (0.2 mm thick, 160 g/m^2^). The origami metamaterials (Fig. 5) were built using card paper (0.12 mm thick, 100 g/m^2^). Cells of three sample units (Fig. 3) and the robotic limb (Fig. 4) were fabricated with polyurethane elastomer (Hei-Cast 8400, 0.6 mm thick, Young’s modulus, 12 MPa). Carbon fiber laminate sheet (0.4 mm thick, Young’s modulus 230 GPa) was bonded to each facet of cells to maintain rigid origami. The flexible air-tight skin is fabricated with polyethylene film (0.15 mm thick). The details about prototype fabrication are provided in texts S3 and S4.

SMA strips (edge length of 32.0 mm, width of 8.0 mm, and thickness of 0.15 mm) of the robotic limb were heating up with a current of 9.5A, power on 5.0 s. The air pump could provide a −90 kPa vacuum with 15 liter/min air flow. The details of lifting and grabbing experiment details of the multistable robotic limb are described in text S4. The mechanical testing of the sample units was carried out on a tensile testing machine INSTRON 9350.

## References

[1] C. Harvey, V. B. Baliga, J. C. M. Wong, D. L. Altshuler, D. J. Inman, Birds can transition between stable and unstable states via wing morphing. Nature 603, 648–653 (2022). 35264798 10.1038/s41586-022-04477-8 PMC8942853

[2] T. Chen, M. Pauly, P. M. Reis, A reprogrammable mechanical metamaterial with stable memory. Nature 589, 386–390 (2021). 33473228 10.1038/s41586-020-03123-5

[3] W. Kim, J. Byun, J. K. Kim, W. Y. Choi, K. Jakobsen, J. Jakobsen, D. Y. Lee, K. J. Cho, Bioinspired dual-morphing stretchable origami. Sci. Robotics 4, eaay3493 (2019). 10.1126/scirobotics.aay3493 33137780

[4] X. Zhang, J. Ma, M. Li, Z. You, X. Wang, Y. Luo, K. Ma, Y. Chen, Kirigami-based metastructures with programmable multistability. Proc. Natl. Acad. Sci. 119, e2117649119 (2022). 35254898 10.1073/pnas.2117649119 PMC8931353

[5] J. A. Faber, A. F. Arrieta, A. R. Studart, Bioinspired spring origami. Science 359, 1386–1391 (2018). 29567709 10.1126/science.aap7753

[6] R. Guseinov, C. McMahan, J. Pérez, C. Daraio, B. Bickel, Programming temporal morphing of self-actuated shells. Nat. Commun. 11, 237 (2020). 31932589 10.1038/s41467-019-14015-2 PMC6957700

[7] R. M. Erb, J. S. Sander, R. Grisch, A. R. Studart, Self-shaping composites with programmable bioinspired microstructures. Nat. Commun. 4, 1712 (2013). 23591879 10.1038/ncomms2666

[8] B. Haghpanah, L. Salari-Sharif, P. Pourrajab, J. Hopkins, L. Valdevit, Multistable shape-reconfigurable architected materials. Adv. Mater. 28, 7915–7920 (2016). 27384125 10.1002/adma.201601650

[9] H. Yasuda, T. Tachi, M. Lee, J. Yang, Origami-based tunable truss structures for non-volatile mechanical memory operation. Nat. Commun. 8, 962 (2017). 29042544 10.1038/s41467-017-00670-w PMC5714951

[10] D. Y. Lee, S. R. Kim, J. S. Kim, J. J. Park, K. J. Cho, Origami wheel transformer: A variable-diameter wheel drive robot using an origami structure. Soft Robotics 4, 163–180 (2017). 29182094 10.1089/soro.2016.0038

[11] D. Y. Lee, J. K. Kim, C. Y. Sohn, J. M. Heo, K. J. Cho, High-load capacity origami transformable wheel. Robotics 6, eabe0201 (2021). 10.1126/scirobotics.abe0201 34043563

[12] J. L. Silverberg, A. A. Evans, L. McLeod, R. C. Hayward, T. Hull, C. D. Santangelo, I. Cohen, Using origami design principles to fold reprogrammable mechanical metamaterials. Science 345, 647–650 (2014). 25104381 10.1126/science.1252876

[13] D. Melancon, B. Gorissen, C. J. García-Mora, C. Hoberman, K. Bertoldi, Multistable inflatable origami structures at the metre scale. Nature 592, 545–550 (2021). 33883736 10.1038/s41586-021-03407-4

[14] K. Kuribayashi, K. Tsuchiya, Z. You, D. Tomus, M. Umemoto, T. Ito, M. Sasaki, Self-deployable origami stent grafts as a biomedical application of Ni-rich TiNi shape memory alloy foil. Mater. Sci. Eng. A 419, 131–137 (2006).

[15] S. Felton, M. Tolley, E. Demaine, D. Rus, R. Wood, A method for building self-folding machines. Science 345, 644–646 (2014). 25104380 10.1126/science.1252610

[16] S. Li, D. M. Vogt, D. Rus, R. J. Wood, Fluid-driven origami-inspired artificial muscles. Proc. Natl. Acad. Sci. U.S.A. 114, 13132–13137 (2017). 29180416 10.1073/pnas.1713450114 PMC5740677

[17] Q. Ze, S. Wu, J. Nishikawa, J. Dai, Y. Sun, S. Leanza, C. Zemelka, L. S. Novelino, G. H. Paulino, R. R. Zhao, Soft robotic origami crawler. Sci. Adv. 8, eabm7834 (2022). 35353556 10.1126/sciadv.abm7834 PMC8967224

[18] S. Wu, Q. Ze, J. Dai, N. Udipi, G. H. Paulino, R. Zhao, Stretchable origami robotic arm with omnidirectional bending and twisting. Proc. Natl. Acad. Sci. U.S.A. 118, e2110023118 (2021). 34462360 10.1073/pnas.2110023118 PMC8433528

[19] Z. Zhakypov, J. Paik, Design methodology for constructing multimaterial origami robots and machines. IEEE Trans. Rob. 34, 151–165 (2018).

[20] C. Zhang, Z. Zhang, Y. Peng, Y. Zhang, S. An, Y. Wang, Z. Zhai, Y. Xu, H. Jiang, Plug and play origami modules with all-purpose deformation modes. Nat. Commun. 14, 4329 (2023). 37468465 10.1038/s41467-023-39980-7 PMC10356792

[21] N. Hu, B. Li, R. Bai, K. Xie, G. Chen, A torsion-bending antagonistic bistable actuator enables untethered crawling and swimming of miniature robots. Research 6, 0116 (2023). 37287890 10.34133/research.0116 PMC10243200

[22] M. Schenk, S. D. Guest, Geometry of Miura-folded metamaterials. Proc. Natl. Acad. Sci. U.S.A. 110, 3276–3281 (2013). 23401549 10.1073/pnas.1217998110 PMC3587190

[23] E. T. Filipov, T. Tachi, G. H. Paulino, Origami tubes assembled into stiff, yet reconfigurable structures and metamaterials. Proc. Natl. Acad. Sci. U.S.A. 112, 12321–12326 (2015). 26351693 10.1073/pnas.1509465112 PMC4603468

[24] J. Cai, D. Xiaowei, Z. Ya, F. Jian, T. Yongming, Bistable behavior of the cylindrical origami structure with Kresling pattern. J. Mech. Des. 137, 061406 (2015).

[25] H. Fang, S. Li, H. Ji, K. W. Wang, Dynamics of a bistable Miura-origami structure. Phys. Rev. E 95, 052211 (2017). 28618514 10.1103/PhysRevE.95.052211

[26] H. Ye, Q. Liu, J. Cheng, H. Li, B. Jian, R. Wang, Z. Sun, Y. Lu, Q. Ge, Multimaterial 3D printed self-locking thick-panel origami metamaterials. Nat. Commun. 14, 1607 (2023). 36959260 10.1038/s41467-023-37343-w PMC10036479

[27] M. Meloni, J. Cai, Q. Zhang, D. Sang-Hoon Lee, M. Li, R. Ma, T. E. Parashkevov, J. Feng, Engineering origami: A comprehensive review of recent applications, design methods, and tools. Adv. Sci. 8, 2000636 (2021).

[28] N. A. Pehrson, D. C. Ames, S. P. Smith, S. P. Magleby, M. Arya, Self-deployable, self-stiffening, and retractable origami-based arrays for spacecraft. AIAA Journal 58, 3221–3228 (2020).

[29] S. Wang, Y. Gao, H. Huang, B. Li, H. Guo, R. Liu, Design of deployable curved-surface rigid origami flashers. Mech. Mach. Thoery 167, 104512–104517 (2022).

[30] S. Wang, P. Yan, H. Huang, N. Zhang, B. Li, Inflatable metamorphic origami. Research 6, 0133 (2023). 37228636 10.34133/research.0133 PMC10204744

[31] J. L. Silverberg, J. H. Na, A. A. Evans, B. Liu, T. C. Hull, C. D. Santangelo, R. J. Lang, R. C. Hayward, I. Cohen, Origami structures with a critical transition to bistability arising from hidden degrees of freedom. Nat. Mater. 14, 389–393 (2015). 25751075 10.1038/nmat4232

[32] L. C. Wang, W. L. Song, Y. J. Zhang, M. J. Qu, Z. Zhao, M. Chen, Y. Yang, H. Chen, D. Fang, Active reconfigurable tristable square-twist origami. Adv. Funct. Mater. 30, 1909087 (2020).

[33] P. P. Pratapa, K. Liu, G. H. Paulino, Geometric mechanics of origami patterns exhibiting Poisson’s ratio switch by breaking mountain and valley assignment. Phys. Rev. Lett. 122, 155501 (2019). 31050524 10.1103/PhysRevLett.122.155501

[34] K. Johnson, V. Arroyos, A. Ferran, R. Villanueva, D. Yin, T. Elberier, A. Aliseda, S. Fuller, V. Iyer, S. Gollakota, Solar-powered shape-changing origami microfliers. Sci. Robot. 8, eadg4276 (2023). 37703382 10.1126/scirobotics.adg4276

[35] Q. Zhang, H. Fang, J. Xu, Programmable stopbands and supratransmission effects in a stacked Miura-origami metastructure. Phys. Rev. E 101, 042206 (2020). 32422700 10.1103/PhysRevE.101.042206

[36] K. Liu, T. Tachi, G. H. Paulino, Invariant and smooth limit of discrete geometry folded from bistable origami leading to multistable metasurfaces. Nat. Commun. 10, 4238 (2019). 31530802 10.1038/s41467-019-11935-x PMC6748981

[37] D. Melancon, A. E. Forte, L. M. Kamp, B. Gorissen, K. Bertoldi, Inflatable origami: Multimodal deformation via multistability. Adv. Funct. Mater. 32, 2201891 (2022).

[38] B. H. Hanna, J. M. Lund, R. J. Lang, S. P. Magleby, L. L. Howell, Waterbomb base: A symmetric single-vertex bistable origami mechanism. Smart Mater. Struct. 23, 094009 (2014).

[39] Z. Zhai, Y. Wang, H. Jiang, Origami-inspired, on-demand deployable and collapsible mechanical metamaterials with tunable stiffness. Proc. Natl. Acad. Sci. U.S.A. 115, 2032–2037 (2018). 29440441 10.1073/pnas.1720171115 PMC5834720

[40] L. H. Dudte, E. Vouga, T. Tachi, L. Mahadevan, Programming curvature using origami tessellations. Nat. Mater. 15, 583–588 (2016). 26808459 10.1038/nmat4540

[41] H. Yasuda, J. Yang, Reentrant origami-based metamaterials with negative Poisson’s ratio and bistability. Phys. Rev. Lett. 114, 185502 (2015). 26001009 10.1103/PhysRevLett.114.185502

[42] G. Wang, J. Wang, Y. Yao, F. Yang, H. Yue, Research on programmable spatial capture mechanism and its motion characteristics based on origami principle. Mech Mach. Thoery 181, 105179 (2023).

[43] S. P. Timoshenko, J. M. Gere, *Theory of elastic stability* (Courier Corporation, 2009).

