# Supplementary material for: Reconfigurable origami-inspired multistable metamorphous structures
Source: Sci Adv. 2024 May 29;10(22):eadk8662. doi: 10.1126/sciadv.adk8662 (PMC11135397; doi:10.1126/sciadv.adk8662)
Supplement: Supplementary file 1 — Supplementary Text Figs. S1 to S16 Tables S1 to S3 Legends for movies S1 to S6 [file sciadv.adk8662_sm.pdf]

Science Advances
AAAS

Supplementary Materials for

Reconfigurable origami-inspired multistable metamorphous structures

Chunlong Wang *et al.*

Corresponding author: Hongwei Guo, guohw@hit.edu.cn; Zhong You, zhong.you@eng.ox.ac.uk

Sci. Adv. **10**, eadk8662 (2024)
DOI: 10.1126/sciadv.adk8662

The PDF file includes:

Supplementary Text
Figs. S1 to S16
Tables S1 to S3
Legends for movies S1 to S6

Other Supplementary Material for this manuscript includes the following:

Movies S1 to S6

Supplementary text

SI. Unit cell geometry

Crease patterns of cells are shown in Fig. S1(A) and S1(B) in which the geometrical parameters are a, b and angle α, β. Both cells are rigid origami and could fold into shapes shown in Fig. S1(C) and S1(D), respectively. If θ is taken as the input, the rest of dihedral angles for the cell I marked in the diagrams can be obtained as follows.

$$\cot(\frac{\zeta_{\mathrm{I}}}{2}) = \tan\alpha\cos(\frac{\theta_{\mathrm{VI}}}{2}) \tag{S1}$$

$$\cos\varphi_{\mathrm{I}} = \frac{4(\cos\theta_{\mathrm{VI}}+1)}{4-(\cos\theta_{\mathrm{VI}}+1)(\cos(2\alpha)-1)}-1 \tag{S2}$$

$$\begin{cases} \tan\frac{\delta_{\mathrm{I}}}{2} = \cos\alpha\tan\frac{\theta_{\mathrm{VI}}}{2}, & (0\le\theta\le\pi) \\ \delta_{\mathrm{I}} = \pi, & (\pi<\theta\le 2\pi) \end{cases} \tag{S3}$$

$$\begin{cases} \gamma_{\mathrm{I}} = 2\zeta_{\mathrm{I}}-\pi, & (0\le\theta\le\pi) \\ \gamma_{\mathrm{I}} = \pi, & (\pi<\theta\le 2\pi) \end{cases} \tag{S4}$$

$$\begin{cases} \theta_{\mathrm{VI}} = \theta, & (0\le\theta\le\pi) \\ \theta_{\mathrm{VI}} = 2\pi-\theta, & (\pi<\theta\le 2\pi) \end{cases} \tag{S5}$$

Similarly, for cell II, we have the following.

$$\cot(\frac{\zeta_{\mathrm{II}}}{2}) = \tan\alpha\cos(\frac{\theta_{\mathrm{VII}}}{2}) \tag{S6}$$

$$\cos\varphi_{\mathrm{II}} = \frac{4(\cos\theta_{\mathrm{VII}}+1)}{4-(\cos\theta_{\mathrm{VII}}+1)(\cos(2\alpha)-1)}-1 \tag{S7}$$

$$\begin{cases} \delta_{\mathrm{II}} = \pi, & (0\le\theta\le\pi) \\ \tan\frac{\delta_{\mathrm{II}}}{2} = \cos\alpha\tan\frac{\theta_{\mathrm{VII}}}{2}, & (\pi<\theta\le 2\pi) \end{cases} \tag{S8}$$

$$\begin{cases} \gamma_{\mathrm{II}} = \pi, & (0\le\theta\le\pi) \\ \gamma_{\mathrm{II}} = 2\zeta_{\mathrm{II}}-\pi, & (\pi<\theta\le 2\pi) \end{cases} \tag{S9}$$

$$\begin{cases} \theta_{\mathrm{VII}} = \theta, & (0\le\theta\le\pi) \\ \theta_{\mathrm{VII}} = 2\pi-\theta, & (\pi<\theta\le 2\pi) \end{cases} \tag{S10}$$

If $a = b = 15$ and $\alpha = \beta = \pi/4$, the variations of these angles with respect to θ are plotted in Fig.S2.

Now consider the vertices on the edge of both cells. We can obtain the coordinates of these vertices on edge contours in a Cartesian coordinate system shown in Fig. S1(E) where its origin is at the centre of the pattern. Note that $0 \leq \theta \leq \pi$.

$$\begin{bmatrix} \mathrm{W}_{\text{I-}x} \\ \mathrm{W}_{\text{I-}y} \\ \mathrm{W}_{\text{I-}z} \end{bmatrix} = \begin{bmatrix} a + a\sin(\gamma_{\mathrm{I}}/2) + b/\tan\alpha \\ b\sin(\theta_{\mathrm{VI}}/2) \\ -a\cos(\gamma_{\mathrm{I}}/2) - b\cos(\theta_{\mathrm{VI}}/2) \end{bmatrix} \tag{S11}$$

$$\begin{bmatrix} \mathrm{Q}_{\text{I-}x} \\ \mathrm{Q}_{\text{I-}y} \\ \mathrm{Q}_{\text{I-}z} \end{bmatrix} = \begin{bmatrix} a + a\sin(\gamma_{\mathrm{I}}/2) \\ 0 \\ -a\cos(\gamma_{\mathrm{I}}/2) \end{bmatrix} \tag{S12}$$

$$\begin{bmatrix} \mathrm{W}_{\text{II-}x} \\ \mathrm{W}_{\text{II-y}} \\ \mathrm{W}_{\text{II-z}} \end{bmatrix} = \begin{bmatrix} a - a\cos\zeta_{\mathrm{II}} + b/\tan\alpha \\ b\sin(\theta_{\mathrm{VII}}/2) \\ -a\sin\zeta_{\mathrm{II}} - b\cos(\theta_{\mathrm{VII}}/2) \end{bmatrix} \tag{S13}$$

$$\begin{bmatrix} \mathrm{Q}_{\text{II-}x} \\ \mathrm{Q}_{\text{II-y}} \\ \mathrm{Q}_{\text{II-z}} \end{bmatrix} = \begin{bmatrix} a - a\cos\zeta_{\mathrm{II}} \\ 0 \\ -a\sin\zeta_{\mathrm{II}} \end{bmatrix} \tag{S14}$$

The coordinates of these selected vertices $\mathrm{W_I}$, $\mathrm{W_{II}}$, $\mathrm{Q_I}$ and $\mathrm{Q_{II}}$ versus θ are plotted in Fig. S3, respectively. Curves of vertices $\mathrm{W_I}$ and $\mathrm{W_{II}}$ are completely identical, and so are curves of $\mathrm{Q_I}$ and $\mathrm{Q_{II}}$, indicating that edge contours of two cells fold compatibly when the shaded central rhombuses are bonded together. In other words, cells I and II can be assembled into a single origami unit.

The transition process of the origami unit is shown in Fig. S4. $\theta = \pi$ is a kinematic bifurcation point where cells I and II could switch over.

SII. Potential energy analysis

In the analytical model of the origami unit, elasticity is confined to the active creases. In other words, these active creases are rotational joints with a torsional spring whereas facets are regarded as rigid thin plates. In practice, these creases are compliant flexural thin plates with a small width as shown in Fig. S5.

The key assumptions in the analysis are summarised as follows.

(1) Facets are completely rigid.

(2) Material along the active creases is linear elastic.

(3) Deformation along the creases never exceed the elastic range of the material.

Based on the thin plate bending theory, the bending strain energy E_B resulting from the change of rotation angle $\Delta\phi$ can be given as follows:

$$\Delta\phi = \frac{Md}{EI}, \text{ and } I = \frac{Lt^3}{12} \tag{S15}$$

where M is the bending moment of the material, E is the Young's modulus, I is the second moment of inertia of a rectangular cross section, and d is the width, L is the length, t is the thickness of the compliant segment. Hence, the bending strain energy E_B can be obtained by:

$$E_B = \frac{EI}{2d}(\Delta\phi)^2 = \frac{1}{2}\frac{EI}{d}(\Delta\phi)^2 = \frac{1}{2}kL(\Delta\phi)^2 \tag{S16}$$

where

$$k = \frac{EI}{dL} = \frac{Et^3}{12d} \tag{S17}$$

Thus, the creases are assumed to behave like linear elastic torsional springs with a stiffness k per unit length.

Potential energies of cells I and II, $\Pi_{\text{C-I}}$ and $\Pi_{\text{C-II}}$, are

$$\Pi_{\text{C-I}} = \sum_{i=1}^{m}\frac{1}{2}kL_i(\phi_i - \phi_{i0})^2 \tag{S18}$$

$$\Pi_{\text{C-II}} = \sum_{j=1}^{n}\frac{1}{2}kL_j(\phi_j - \phi_{j0})^2 \tag{S19}$$

where m and n are total number of creases in each cell, L is the length of a crease, and ϕ and ϕ_0 are current and initial rest dihedral angles of a crease. These angles are related to θ, and ϕ_0 is therefore related the initial state of a cell.

It is noteworthy that the edge creases of origami unit are formed when the edges of two cells are bonded together, and potential energy of edge creases, Π_{E}, can be calculated as

$$\Pi_{\text{E}} = \sum_{g=1}^{p}\frac{1}{2}kL_g(\phi_g - \phi_{g0})^2 \tag{S20}$$

where p is the total number of edge creases in a unit. The rest angles at the edge creases are set as the angles when bonding of the cells takes place.

Thus, the total potential energy of a unit can be expressed as

$$\Pi = \Pi_{\text{C-I}} + \Pi_{\text{C-II}} + \Pi_{\text{E}} \tag{S21}$$

The geometric dimensions and material properties of these units shown in Figure 2 are identical, which are listed in Table S1.

Considering cell I with a rest angle $\theta_{I0} = \pi/2$, parameters of the initial state can be calculated $\theta_{VI0} = \pi/2$, $\delta_{I0} = 0.392\pi$, $\varphi_{I0} = 0.392\pi$. When cell II with a rest angle $\theta_{II0} = 3\pi/2$, parameters of the initial state are $\theta_{VII0} = \pi/2$, $\delta_{II0} = 0.392\pi$, $\varphi_{II0} = 0.392\pi$. The initial rest angles at the edge creases are $\psi_0 = 0.392\pi$. Based on equations (S18) - (S21), we plot normalised potential energy vs. θ in Figures 2(A) and 2(B).

When cell I with a rest angle $\theta_{I0} = \pi/3$, parameters of the initial state are $\theta_{VI0} = \pi/3$, $\delta_{I0} = 0.247\pi$, $\varphi_{I0} = 0.247\pi$. When cell I with a rest angle $\theta_{I0} = 2\pi/3$, parameters of the initial state are $\theta_{VI0} = 2\pi/3$, $\delta_{I0} = 0.564\pi$, $\varphi_{I0} = 0.564\pi$. Then, we can get the normalised potential energy landscapes of units shown in Figures 2(C) and 2(D), respectively.

As indicated in Fig. S4(C), it is theoretically possible both cells deploy to an identical shape after passing the bifurcation state, however, this never occurred in practice when creases are elastic. The proof is as follows.

Considering the origami unit with $\theta_{I0} = \pi/2$ and $\theta_{II0} = 3\pi/2$, Fig. S6(A) shows the normalised potential energies of four possible transition modes for each cell, which are denoted as $\Pi_{C\text{-}I1}$, $\Pi_{C\text{-}I2}$ for cell I, and $\Pi_{C\text{-}II1}$, $\Pi_{C\text{-}II2}$ for cell II, respectively. There would be three cases:

(1) Cells I and II switch over after passing the bifurcation state, the total potential energy of a unit $\Pi_1 = \Pi_{C\text{-}I1} + \Pi_{C\text{-}II1} + \Pi_{E1}$.

(2) Cell II deploys to an identical shape of cell I after passing the bifurcation state, the total potential energy of a unit $\Pi_2 = \Pi_{C\text{-}I1} + \Pi_{C\text{-}II2} + \Pi_{E2}$.

(3) Cell I deploys to an identical shape of cell II after passing the bifurcation state, the total potential energy of a unit $\Pi_3 = \Pi_{C\text{-}I2} + \Pi_{C\text{-}II1} + \Pi_{E3}$.

Figure S6(B) shows the total potential energies of these three cases. Obviously, it requires higher NPE when two cells deploy into an identical shape, which indicates this will never happen.

SIII. Experimental investigation

1. Fabrication of prototypes

Using 3D-printed moulds, cells were casted using polyurethane elastomer (Hei-Cast 8400, shore 80A, Young's modulus 12 Mpa). Each cell is manufactured at the initial unstrained shapes and edge creases are assigned in the cell II, as shown in Fig. S7. The thicknesses of all elastomeric facets and sections of active creases are 0.6mm. All cells in their stable configurations are shown in Table S2. Rigid plates, cut from 0.4mm thick carbon fibre laminates (Young's modulus 230 Gpa), were then

attached to facets of the cells before the cells were bonded together. Three unit prototypes were made, which are shown in Figure 3(A).

2. Assembly of the unit samples

There were three steps to construct a unit: manufacturing preliminary cells (casting cells and then attaching carbon fibre laminate facets to the cells, as shown in Fig. S8), deploying cell II to a stable configuration 2 in Table S2 while keeping cell I in stable configuration 1, and then bonding two cells together. In the final process, two central rhombuses of both cells are glued together and the 4.0 mm wings around the edges of cells were used to bond two cells together along the edges. Since the two central rhombuses are rather thick in comparison with other facets, slits were made along the diagonals of two central rhombuses to reduce the influence of thickness.

A rig consisting of a pair of holding moulds was made, as shown in Fig. S9, to assemble the cells more accurately. It was used to hold both cells together prior to their being bonded together to create a unit.

3. Mechanical testing of unit

To measure the force required to keep a unit at an unstable equilibrium shape, we carried out the experiments using a tensile testing instrument (INSTRON 9350), as shown in Fig. S10.

Because of the transition between two stable configurations, the whole test regime of a unit was divided into four stages and test methods were designed according to their folding behaviours, as shown in Fig. S11. The unit was placed horizontally on the support base when it was folded to $\theta = \pi$ from the stable equilibrium positions, as shown in Fig. S11(A). On the contrary, it was placed upright when it was folded to $\theta = 0$, as shown in Fig. S11(B). These arrangements made it easy to collect data. Four tests were carried out for an origami unit in total.

The data from the four tests were combined into a single plot in Figure 3C, and thus the strain energy of an origami unit can be obtained by the integration of the measured forces over the deformation, which can be expressed as:

$$E = \int_0^{S_T} f(s)ds, \tag{S22}$$

where $f(s)$ is the measured force, S_T is the compression displacement. For the force data was collected discreetly with the same displacement increment, the integration given in Eq. (S22) can be simplified to

$$E = \Delta s\left[f(s_1) + f(s_2) + ... + f(s_n)\right], \tag{S23}$$

where Δs is the displacement increment, and $f(s)$ is force at displacement s.

SIV. Multistable robotic limb

As shown in Figure 4(B), a pair of arc-shaped SMA strips were symmetrically installed on either side of the central vertex to facilitate the transition between stable configurations. Figure S12 (A) shows the overview of a SMA actuated unit. When the SMA strip-b is heated, the unit snaps from one stable configuration to the other. Figures S12(B) and (C) show either side of the unit.

The multistable robotic limb consists of three main components: the SMA activated origami structure skeleton, a flexible air-tight skin and a pump to generate pneumatic drive to alter its shape. The skin is sealed as a bag covering the skeleton and the internal space between the skeleton and skin is filled by air. Figure S13 shows the overall view of the multistable robotic limb.

In the experiments, we used the robotic limb to lift and grab objects, including a ping pong ball (diameter 40cm, weight 2.9g), a Rubik's cube (weight 20g), a plastic bottle and an aluminium bottle (diameter 50cm, weight 15g).

SV. Reprogrammable mechanical metamaterials

1. Poisson's ratio

An origami metamaterial is built by assembling a 3 × 7 array of the origami unit (Figs. 5A and 5C), the crease pattern is shown in Fig. S14. The Poisson's ratio of the metamaterial can be defined as:

$$\nu_{xy} = -\frac{(dy/y)}{(dx/x)}, \text{ and } \nu_{zy} = -\frac{(dy/y)}{(dz/z)}, \tag{S24}$$

Based on the geometry described in SI, these parameters can be obtained by the specific stable configurations of this metamaterial. First, taking a derivative of Eq. (S1), we obtain

$$d\zeta_{\mathrm{I}} = \left(\tan\alpha \sin^2(\frac{\zeta_{\mathrm{I}}}{2}) \sin(\frac{\theta_{\mathrm{VI}}}{2}) \right) d\theta, \tag{S25}$$

For the selected stable configurations shown in Figure 5(A), geometric parameters can be expressed as follows, respectively.

Stable configuration *a*

$$\begin{bmatrix} W_x \\ B_y \\ H_z \end{bmatrix} = \begin{bmatrix} 8\left(a + b/\tan\alpha - a\cos\zeta_{\mathrm{I}}\right) \\ 12b\sin(\theta_{\mathrm{VI}}/2) \\ a\sin\zeta_{\mathrm{I}} + b\cos(\theta_{\mathrm{VI}}/2) \end{bmatrix} \tag{S26}$$

Stable configuration *b*

$$\begin{bmatrix} W_x \\ B_y \\ H_z \end{bmatrix} = \begin{bmatrix} 6(a + b / \tan\alpha) - 8a\cos\zeta_{\mathrm{I}} \\ 12b\sin(\theta_{\mathrm{VI}} / 2) \\ 2(a + b / \tan\alpha)\sin\zeta_{\mathrm{I}} - a\sin(2\zeta_{\mathrm{I}}) \end{bmatrix} \tag{S27}$$

Stable configuration *c*

$$\begin{bmatrix} W_x \\ B_y \\ H_z \end{bmatrix} = \begin{bmatrix} 4a - (6a + 4b / \tan\alpha)\cos\zeta_{\mathrm{I}} + 2b / \tan\alpha \\ 12b\sin(\theta_{\mathrm{VI}} / 2) \\ 2a\sin\zeta_{\mathrm{I}} - 2(a + b / \tan\alpha)\sin(2\zeta_{\mathrm{I}}) + b\cos(\theta_{\mathrm{VI}} / 2) \end{bmatrix} \tag{S28}$$

Stable configuration *d*

$$\begin{bmatrix} W_x \\ B_y \\ H_z \end{bmatrix} = \begin{bmatrix} 6a - 4(2a + b / \tan\alpha)\cos\zeta_{\mathrm{I}} + 2a\cos(2\zeta_{\mathrm{I}}) + 4b / \tan\alpha \\ 12b\sin(\theta_{\mathrm{VI}} / 2) \\ (3a + 2b / \tan\alpha)\sin\zeta_{\mathrm{I}} - a\sin(2\zeta_{\mathrm{I}}) + b\cos(\theta_{\mathrm{VI}} / 2) \end{bmatrix} \tag{S29}$$

Stable configuration *e*

$$\begin{bmatrix} W_x \\ B_y \\ H_z \end{bmatrix} = \begin{bmatrix} 4a - 8(a + b / \tan\alpha)\cos\zeta_{\mathrm{I}} + 2a\cos(2\zeta_{\mathrm{I}}) \\ 12b\sin(\theta_{\mathrm{VI}} / 2) \\ 4(a + b / \tan\alpha)\sin\zeta_{\mathrm{I}} - 2a\sin(2\zeta_{\mathrm{I}}) \end{bmatrix} \tag{S30}$$

Substituting Eqs. (S25) – (S30) into Eqs. (S24), the Poisson's ratio v_{xy} and ratio v_{zy} of this origami metamaterial in the five selected stable configurations were obtained, respectively. The Poisson's ratio was a function of the folding ratio, defined as $(\pi - \theta) / \pi \times 100\%$, where θ is the indicator parameter of a specific configuration (Figures 5B and 5C).

2. Experimental setup

We fabricated a reprogrammable origami metamaterial shown in Figure 5A using paper cards. Key geometry parameters are defined with $a = b = 15$ and $\alpha = \beta = \pi/4$. To obtain compression configuration of the metamaterial when it was compressed in the y direction, an acrylic plate was placed on the top surface of the sample for loading, as shown in Fig. S15 (the final compression load was 0.4kg).

3. Polygonal shapes

Figure 5(E) shows a single origami structure made from 11 units. Amongst its 55 distinct configurations, there exist polygonal shapes, some of which are shown in Fig. S16. Using kinematic relationships in Eq. (S1), we can obtain θ for each polygonal shape when the first unit meets the last unit. These angles are given in Table S3.

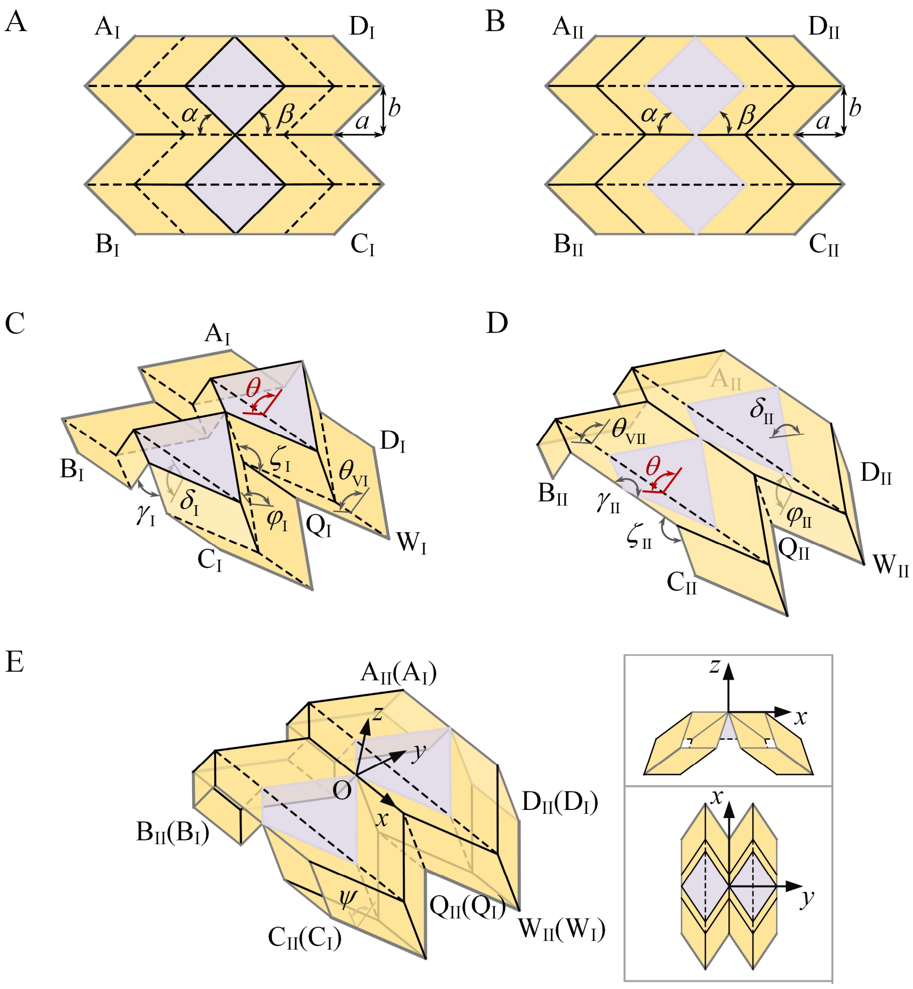

Fig. S1. Schematic and geometry of cells in the origami unit. (**A**) The crease pattern for cell I on a flat sheet, in which black solid lines act as mountain folds and dashed lines are valley creases. (**B**) The crease pattern for cell II. (**C**) The folded shape of cell I with key geometric parameters (axonometric). (**D**) The folded shape of cell II with key geometric parameters (axonometric). (**E**) The origami unit created by bonding cells together, which is a rigid, flat deployable structure (axonometric).

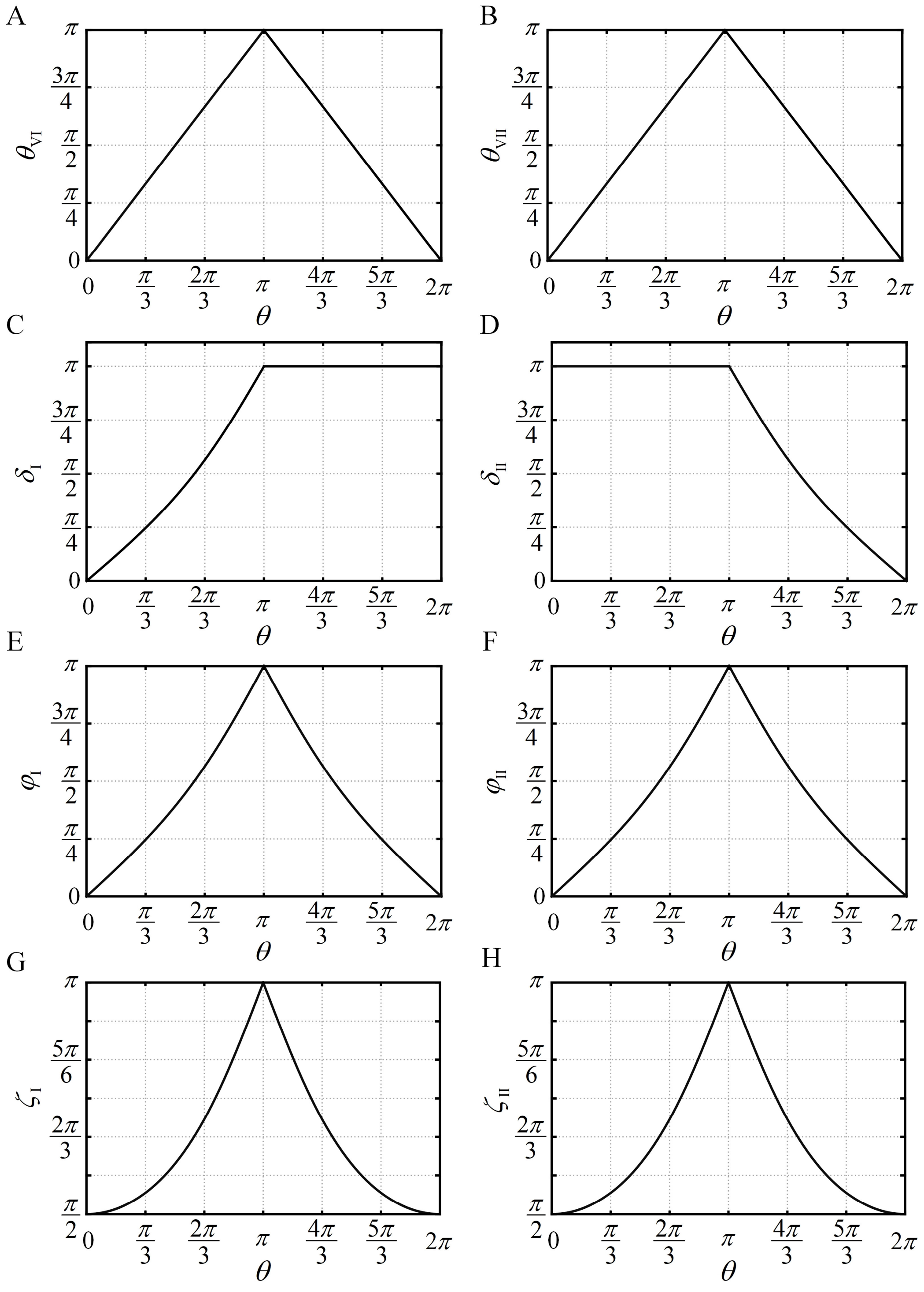

Fig. S2. Kinematic behaviour of cells. (**A**) θ_{VI} versus θ in cell I. (**B**) θ_{VII} versus θ in cell II. (**C**) δ_{I} versus θ in cell I. (**D**) δ_{II} versus θ in cell II. (**E**) φ_{I} versus θ in cell I. (**F**) φ_{II} versus θ in cell II. (**G**) ζ_{I} versus θ in cell I. (**H**) ζ_{II} versus θ in cell II.

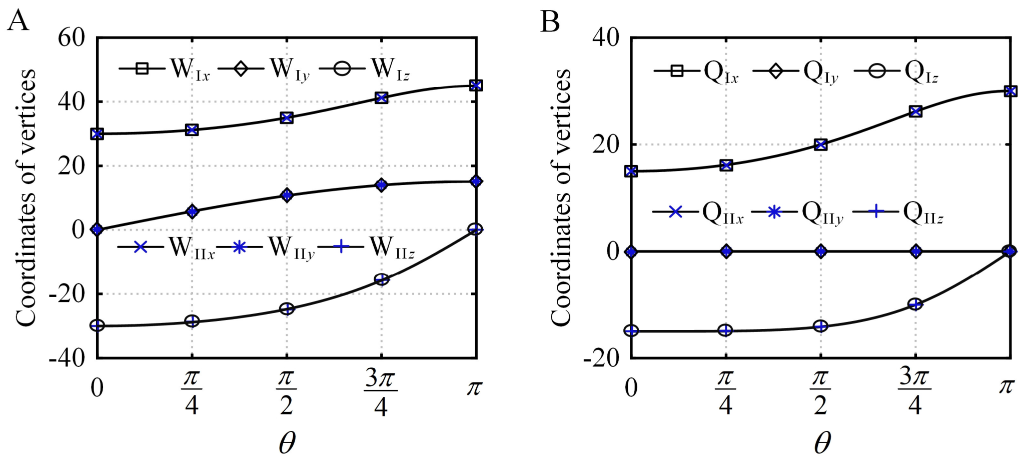

Fig. S3. Coordinates of selected vertices. (**A**) W_I in cell I and W_{II} in cell II. (**B**) Q_I in cell I and Q_{II} in cell II

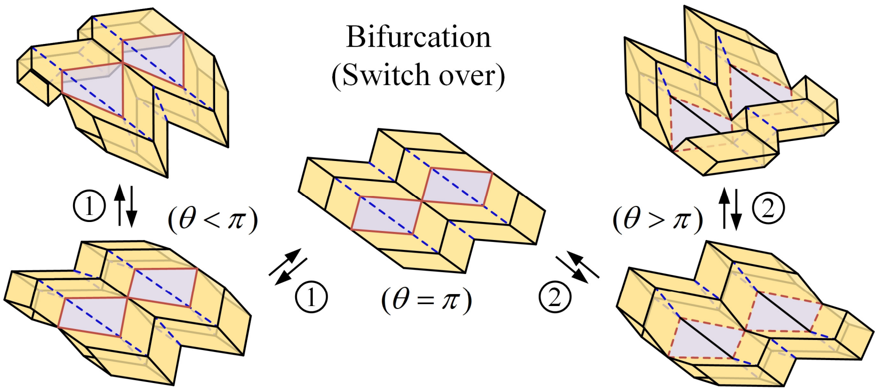

Fig. S4. Transition process of origami unit. Both cells are completely flat when $\theta = \pi$, and it is the kinematic bifurcation point where cell I and cell II are switched over.

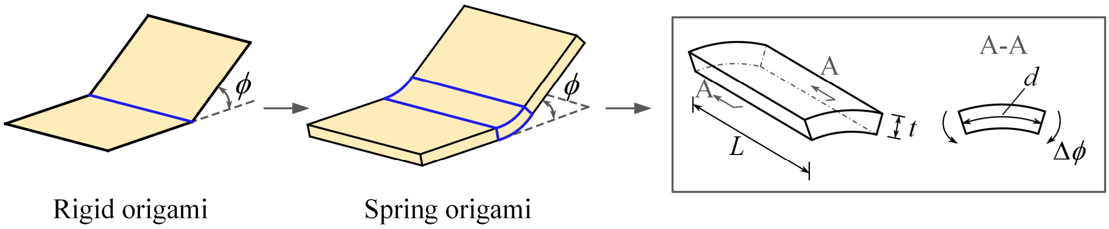

Fig. S5. Parameters used to define small-width flexural thin plates. The rotation $\Delta\phi$ is described by the plate bending in the crease.

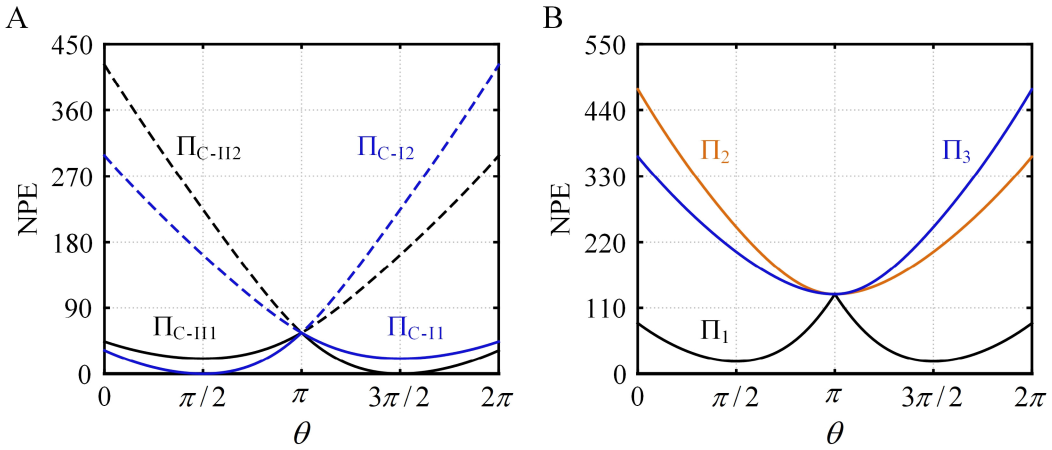

Fig. S6. Potential energy of possible transition modes for the origami unit. (**A**) NPE for cells. (**B**) NPE for unit.

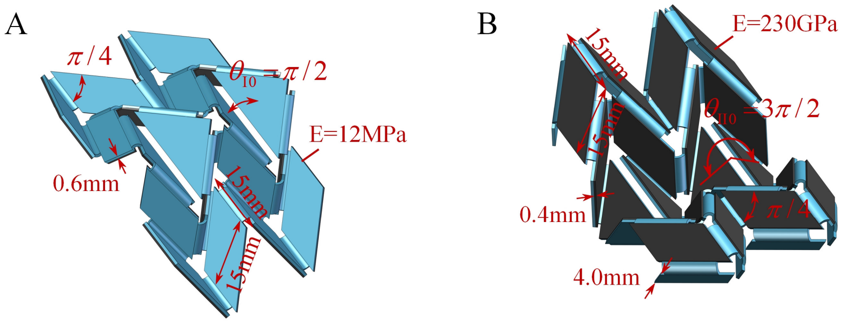

Fig. S7. Geometry and material properties for cells at initial unstrained configurations. (**A**) Cell I with $\theta_{\mathrm{I}0} = \pi/2$. (**B**) Cell II with $\theta_{\mathrm{II}0} = 3\pi/2$.

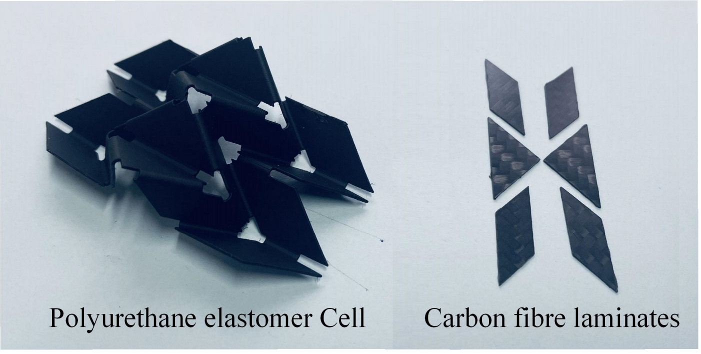

Fig. S8. PU cells and carbon fibre laminates used to construct cells in Table S2. The carbon fibre laminates were bonded with ergo.5800 glue.

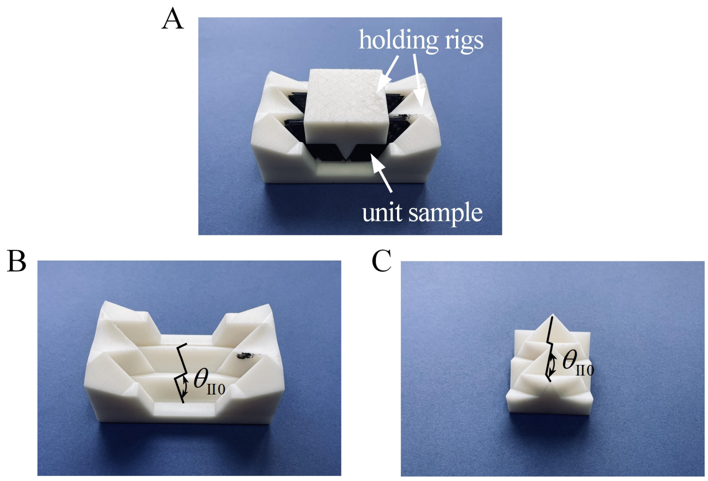

Fig. S9. The holding rig. (**A**) The holding rig that holds two cells together. (**B**) Female mould. (**C**) Male mould.

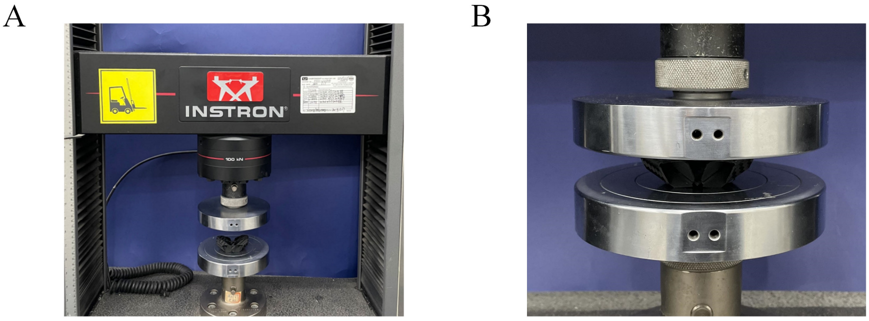

Fig. S10. Experiment setup. (**A**) INSTRON 9350 used for the test. (**B**) A unit placed for testing.

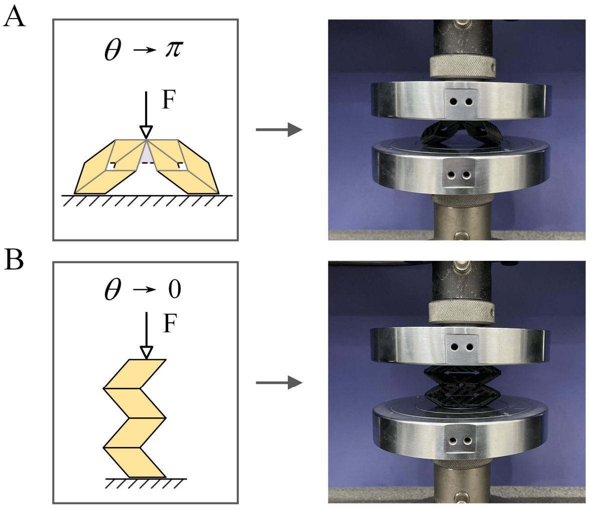

Fig. S11. Loading regimes. (**A**) The unit is folded to $\theta = \pi$. (**B**) The unit is folded to $\theta = 0$ from the stable equilibrium configurations.

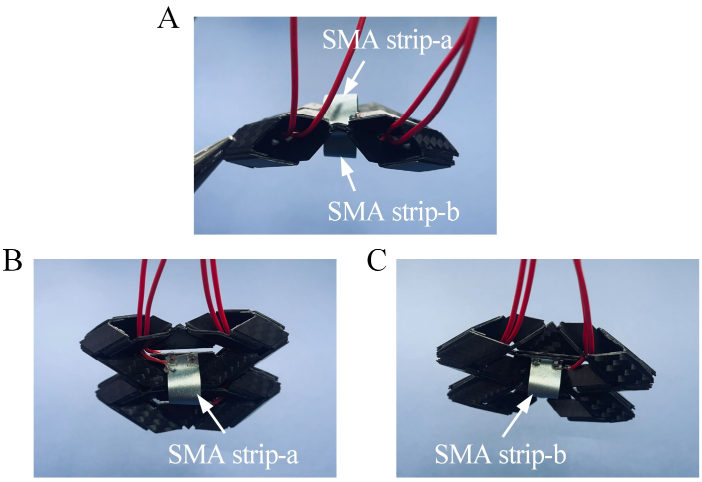

Fig. S12. Arrangements of two SMA strips in an origami unit. (**A**) Side view. (**B**) Top view. (**C**) Bottom view.

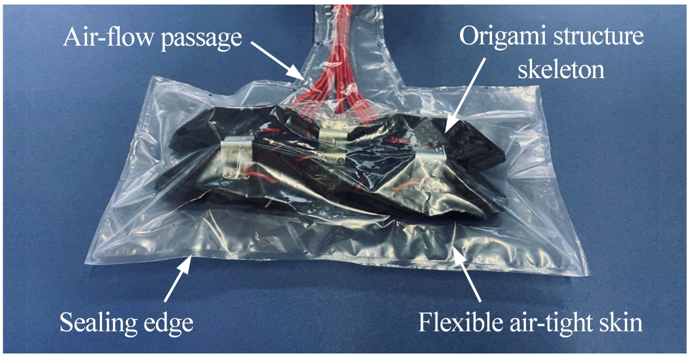

Fig. S13. Overall view of the multistable robotic limb. The flexible air-tight skin is sealed as a bag covering the SMA actuated origami structure skeleton.

A

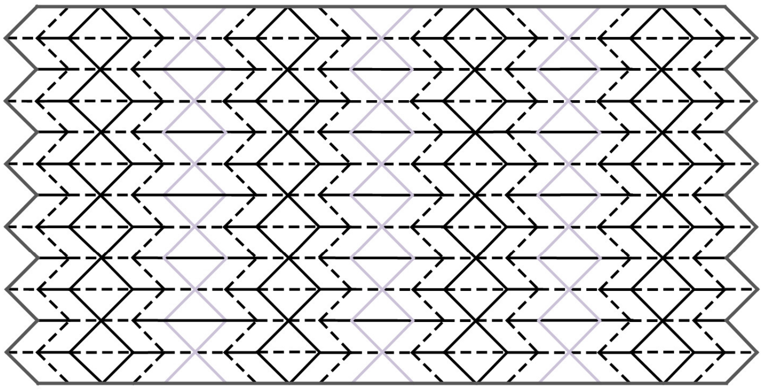

B

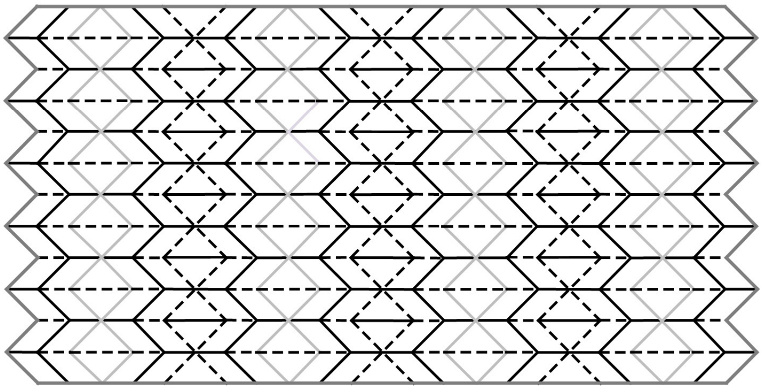

Fig. S14. The crease patterns for cells of the reprogrammable metamaterial on a single sheet in Figure 5A. (**A**) Crease pattern for cell I. (**B**) Crease pattern for cell II. The grey edges of rhombuses are dormant creases

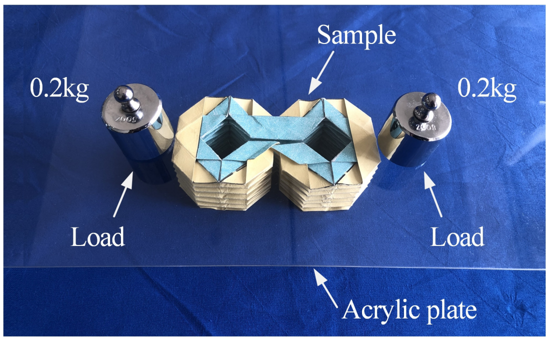

Fig. S15. Compression setup of a metamaterial sample. An acrylic plate was placed on the top surface and the load was applied in the vertical direction.

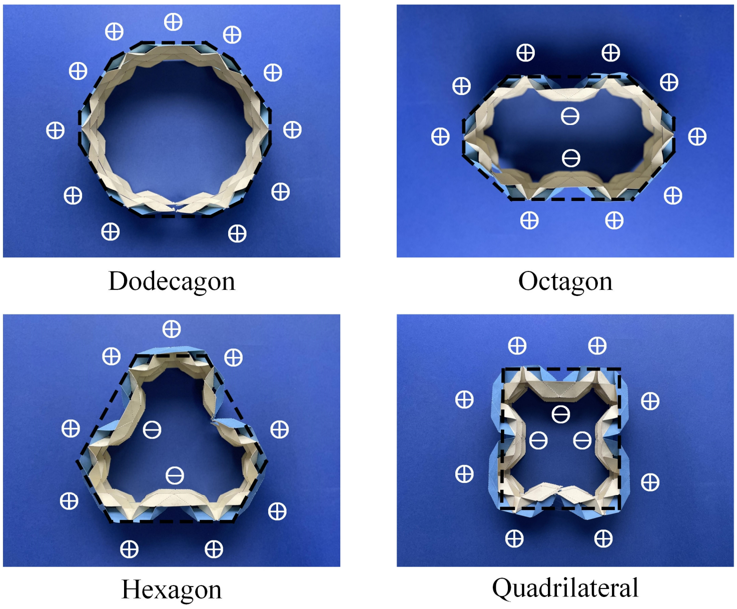

Fig. S16. Polygonal shapes constructed by the origami structure made from 11 units.

Table S1. Geometric dimensions and material properties

a (mm)	*b* (mm)	α	β	*d* (mm)	*t* (mm)	*E* (Mpa)
15	15	π / 4	π / 4	0.7	1.0	12.0

Table S2. Stable configurations of cells

	stable configuration 1	stable configuration 2
<u>Cell I</u> $\theta_{I0} = \pi/3$	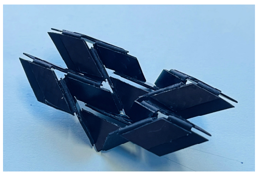	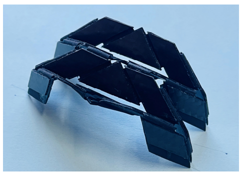
<u>Cell I</u> $\theta_{I0} = \pi/2$	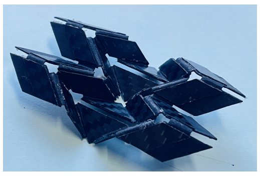	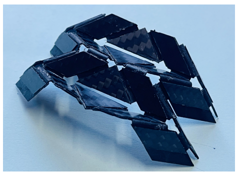
<u>Cell I</u> $\theta_{I0} = 2\pi/3$	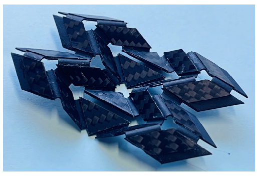	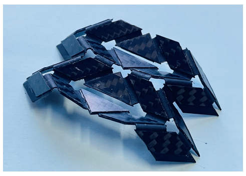
<u>Cell II</u> $\theta_{II0} = 3\pi/2$	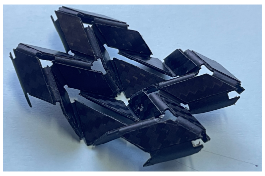	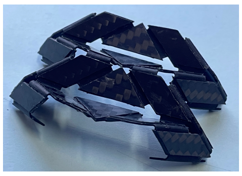

Table S3. θ for polygonal shapes

Dodecagon	$\theta = 0.8273\pi$
Octagon	$\theta = 0.7281\pi$
Hexagon	$\theta = 0.6082\pi$
Quadrilateral	$\theta = 0$

Movie S1.

A card bistable origami unit and transition process between different configurations.

Movie S2.

Transition process between two stable configurations of SU1.

Movie S3.

Origami skeleton reconfigures operational configurations.

Movie S4.

The robotic limb lifts a weight.

Movie S5.

The robotic limb grabs objects.

Movie S6.

Reconfigurable and programmable origami metamaterials.
